# Ten-Year Antimicrobial Resistance Trend in Uropathogenic *Escherichia coli* (UPEC) Isolated from Dogs and Cats Admitted to a Veterinary Teaching Hospital in Italy

**DOI:** 10.3390/microorganisms12112175

**Published:** 2024-10-29

**Authors:** Alessandro Bellato, Patrizia Robino, Maria Cristina Stella, Daniela Scalas, Paolo Savarino, Renato Zanatta, Giovanni Re, Patrizia Nebbia

**Affiliations:** 1 Department of Veterinary Sciences, University of Turin, Largo Paolo Braccini 2, 10095 Grugliasco, Italy; alessandro.bellato@unito.it (A.B.); patrizia.robino@unito.it (P.R.); mariacristina.stella@unito.it (M.C.S.); paolo.savarino@unito.it (P.S.); renato.zanatta@unito.it (R.Z.); giovanni.re@unito.it (G.R.); 2 Blood Component Production and Validation Center, University Hospital Città della Salute e della Scienza di Torino, 10126 Turin, Italy; dscalas@cittadellasalute.to.it

**Keywords:** uropathogenic *Escherichia coli* (UPEC), antimicrobial resistance (AMR), dog, cat, susceptibility testing

## Abstract

Urinary tract infections (UTIs) are a common occurrence in cats and dogs. Surveillance of antibiotic resistance trends helps in the prudent selection of suitable antimicrobial agents. However, there are limited available data on this matter in Italy. This retrospective study aimed to investigate the trends of antimicrobial resistance in uropathogenic *Escherichia coli* (UPEC) isolated from cats and dogs over ten years (January 2014 to October 2023). Three hundred thirty-nine UPEC strains were isolated from urine samples submitted to the Veterinary Teaching Hospital of Torino (Italy). Antimicrobial susceptibility testing was conducted for up to 11 classes of antibacterials, categorized into four categories (A, B, C, and D) following the European Medicine Agency guidelines for prudent antimicrobial use in animals. The results reveal a higher resistance towards compounds in categories C and D, while fewer isolates were resistant to antibacterials in categories B and A. Resistance has steadily increased from 2014 to 2019. Starting from 2020, a decline in resistance is evident in all four categories. The reduction is more pronounced for the categories subject to the greatest restrictions under European and national legislation. The change in resistance trend is in line with findings from other European countries and food-production animals in Italy.

## 1. Introduction

In companion animals, extra-intestinal pathogenic *Escherichia coli* (ExPEC) can cause a variety of diseases [1]. Uropathogenic *Escherichia coli* (UPEC) is a subcategory of ExPEC responsible for 33–51% of all canine and feline urinary tract infection (UTI) cases [2,3,4,5,6,7,8]. UTIs are more common in dogs, with a reported prevalence of around 14% [9], than in cats, where the observed prevalence ranges from 1 to 8% [10,11,12]. Companion animals and their owners share some common UPEC strains, and several studies confirmed their zoonotic potential, evidencing that isolates from dogs can invade human bladder cells, induce cytotoxicity, and activate the synthesis of proinflammatory cytokines like human isolates [13]. UTIs lead to discomfort, urinary tract complications, and potentially life-threatening conditions if left untreated [14,15]. Recommended first-line antimicrobials for uncomplicated UTIs in dogs and cats include amoxicillin, amoxicillin-ac. clavulanic, or trimethoprim-sulphonamide [16]. However, these recommendations might not apply to different geographical locations due to different antimicrobial susceptibility patterns, making antimicrobial susceptibility testing (AST) instrumental in providing adequate treatment [17]. Moreover, the utilization of those recommended antimicrobials in companion animals over the past decade has been associated with a concerning increase in antimicrobial resistance (AMR) among pathogenic urinary bacteria, particularly *E. coli* isolates [5,18]. Indeed, studies reported that AMR has rendered the management of UTIs more intricate, as the multi-drug resistant (MDR) strains exceed 50% [1,19].

To address the critical issue of AMR, the European Medicines Agency (EMA) created a specific working group (Antimicrobial Advice Ad Hoc Expert Group, AMEG) [20]. The AMEG established a categorization system that divides antibacterials used in veterinary medicine into distinct groups based on their significance for public health and the potential impact on the development of AMR associated with their use in veterinary practice [20]. It categorizes antibacterials into four categories: A (Avoid), critical importance to human medicine; B (Restrict), high importance to human medicine; C (Caution), moderate importance to human medicine; and D (Prudence), limited importance to human medicine.

EMA recommendations guide the fight against AMR at the European level. In Italy, the National Plan for Combating Antimicrobial Resistance (PNCAR) 2017–2020 and 2022–2025 [21,22] incorporated these recommendations promoting prudent use of antibacterials under a One Health approach to mitigate AMR. In addition, to monitor AMR in food-producing animals, a nationwide system has been implemented using *E. coli* as the AMR marker, thanks to its ability to gather resistance genes [23,24].

A similar AMR monitoring system for companion animals would also be instrumental in adjusting UTI treatment because first-line antibiotic recommendations for UTI are generically formulated and might not apply to locally circulating AMR patterns [17]. Unfortunately, to date, it is not yet available. Regional studies conducted in Italy reported concerning resistance of *E. coli* against various antibacterials. One reported that isolates were mostly resistant to clindamycin (60%), erythromycin (59%), ampicillin (53%), and enrofloxacin (48%) [25]. Another one mentioned ampicillin (48%), cephalotin (39%), cefalexin (32%), and cefpodoxime (31%) [4]. These two examples show the need for a selection process for UTI treatment, considering that AMR patterns might differ even in neighboring areas within the same country [26]. Local studies like this one can help guide antibacterial treatment. Therefore, this retrospective study aimed to evaluate the AMR trends in UPEC isolated from cats and dogs admitted to the Veterinary Teaching Hospital (VTH) of the University of Turin from 2014 to 2023 and provide an indication for UTI treatment.

## 2. Materials and Methods

### 2.1. Study Design

This is a retrospective study, collecting all data on antimicrobial susceptibility testing results of UPEC isolated from clinical urine specimens submitted to the bacteriology laboratory of the Veterinary Teaching Hospital of the University of Turin from January 2014 to October 2023. The VTH catching area, concentrated in the province of Turin, includes all of the Piedmont region in northwestern Italy.

### 2.2. Sample Collection and Bacteriological Procedures

Informed consent of the owner was regularly acquired prior to performing any clinical procedure. Urine samples were collected from dogs and cats by ultrasound-guided cystocentesis for clinical diagnostics purposes. Routinary, 10 uL of sample are inoculated onto Columbia agar plates with 5% sheep blood (COL+SB, Oxoid, Basingstoke, UK) and aerobically incubated at 37 °C for 24 h for quantitative evaluation of bacteriuria (limit of detection, LOD = 10^2^ CFU/mL). Plates negative after 24 h are read at 48 h for slow-growing bacteria. Before 2019, *E. coli* identification was based on Gram-staining, colony morphology on MacConkey agar (MCC, Oxoid, Basingstoke, UK), lactose fermentation, and reaction on triple sugar iron agar (TSI, Oxoid, Basingstoke, UK) [27]. Since 2019, isolates have been identified using matrix-assisted laser desorption/ionization time-of-flight (MALDI ToF) mass spectrometry, following the manufacturer recommendations (MALDI Biotyper, Bruker Daltonics, Bremen, Germany), which indicate confident identification at species level with scores > 1.99.

### 2.3. Antimicrobial Susceptibility Testing

Antimicrobial susceptibility testing (AST) was performed by the agar disk diffusion method (ADD), following the protocol of CLSI Vet [28]. The diameter of the zone of inhibition was measured to interpret the susceptibility pattern in accordance with veterinary CLSI guidelines [29]. Human breakpoints were employed in instances when veterinary breakpoints were not available [30].

Eleven classes of antibacterials were tested: aminoglycosides, aminopenicillins with beta-lactamase inhibitors, aminopenicillins without beta-lactamase inhibitors, amphenicols, carbapenems, first and second-generation cephalosporins, third and fourth-generation cephalosporins, nitrofuran derivatives, quinolones, sulphonamides with or without folate-reductase inhibitors, and tetracyclines. The antibacterial classes employed in this study included those which isolates are usually tested for. Some antibacterials represent common UTI treatment (e.g., ampicillin, amoxicillin-clavulanic acid, etc.), while others were tested by means of passive surveillance even though their use is restricted or forbidden in veterinary medicine (e.g., carbapenems, piperacillin, fosfomycin, tigecycline). They were chosen in accordance with EMA categorization and AMEG guidelines for antibacterial treatment in veterinary medicine (Table S1). Occasionally, certain classes were not tested, hence the number of isolates tested for each antibacterial may differ.

### 2.4. Data Analysis

AMR prevalence was estimated including all laboratory reports of UPEC from January 2014 to October 2023. Since tested antibacterial agents have changed during the study period, data were aggregated by antibacterial class. Isolates susceptible to increased dosage were considered susceptible, as recommended by the European Committee on Antimicrobial Susceptibility Testing (EUCAST) guidelines [31]. Age, sex, and host species were evaluated as potential confounders for antimicrobial resistance by a multivariate logistic regression for each ith antibacterial class:

(1) $$\ln\left(\frac{\pi_{i}}{1-\pi_{i}}\right)=\beta_{0,i}+\beta_{1,i}age+\beta_{2,i}sex+\beta_{3,i}host+\varepsilon_{i},$$

where $ln\left(\frac{\pi_{i}}{1-\pi_{i}}\right)$ was the natural logarithm of the odds of the resistant (R) outcome; β_0_ was the intercept; β_1–3_ was the coefficients of variation by age, sex (reference group: female), and host species (ref. group: dog), respectively; and ε was the residual error [32].

The resistance to aminoglycosides, aminopenicillins with and without beta-lactamase inhibitors, carbapenems, cephalosporins of all generations, quinolones, and sulphonamides was studied throughout the ten-year period. Resistance to amphenicols, nitrofuran derivatives, and tetracyclines was studied from 2020 to 2023 instead because these antibacterials were lately introduced in the AST. Resistance to phosphomycins was tested between 2014 and 2016.

The prevalence of resistant UPEC was visually inspected for changes in the trend. When no change was observed, the trend slope was estimated by means of logistic regression (2).

(2) $$\ln\left(\frac{\pi_{i}}{1-\pi_{i}}\right)=\beta_{0,i}+\beta_{1,i}year+\varepsilon_{i}.$$

For the classes that had a flexion in the trend, a spline with one node was introduced, as shown in (3):

(3) $$\ln\left(\frac{\pi_{i}}{1-\pi_{i}}\right)=\beta_{0,i}+\beta_{1,i}year+\beta_{x,i}x+\varepsilon_{i},$$

where x was alternatively 0 for the year before the trend changed, or the difference between the current year and the year when the trend changed, with β_x,i_ being its coefficient.

Resistance prevalence to EMA categories was calculated for each isolate based on the number of tested antibacterials of a certain category (Table S1). The resistance to EMA categories was visually inspected for changes in the trend, and a regression with one node was employed to estimate the trend slope before and after the flexion, as formally described in (4):

(4) $$\ln\left(\frac{\pi_{j}}{1-\pi_{j}}\right)=\beta_{0,j}+\beta_{1,j}year+\beta_{x,j}x+\varepsilon_{j}$$

Confidence intervals were calculated with a confidence of 95% (95%CI) using Wald’s approximation. Data were analyzed using R version 4.3.2 [33].

## 3. Results

**Figure 1:**
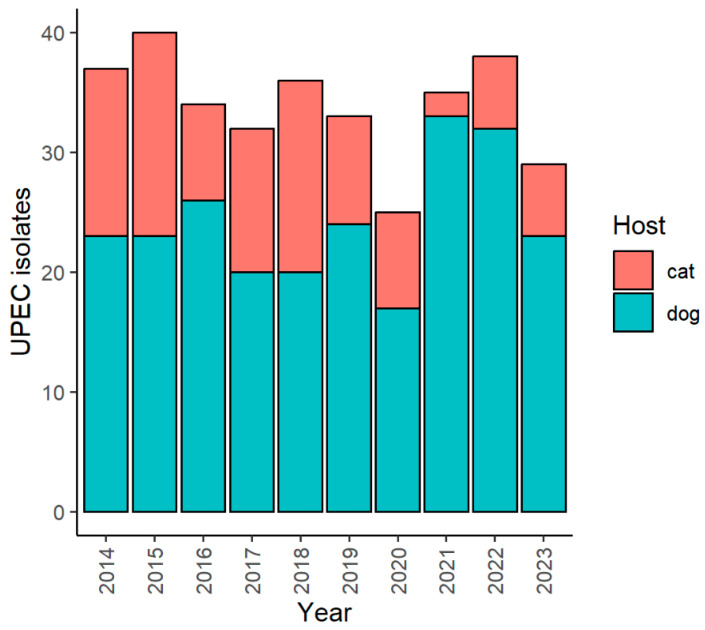
Number of UPEC isolated from dogs and cats per year.

Between 2014 and 2023, a median of 34.5 UPEC isolates was collected per year. In 2020, when the COVID-19 pandemic hit Italy, the hospital always remained open, as pet owners were granted permission to bring their animals despite the lockdown. Nevertheless, a non-significant (*p* = 0.437) reduction in the number of cases was observed (Figure 1).

A grand total of 339 UPEC isolates were collected from 241 (71.1%) dogs, 53.1% of which were males, and 98 (28.9%) cats, of which 63.3% were males. The median dogs’ age was 10 years (min = 0, max = 18), and the median cats’ age was 8 years (min = 0, max = 20.7). Age, sex, and the host species were not associated with resistance to any antibacterial.

### 3.1. Antimicrobial Resistance to Antibacterial Classes

The highest overall resistance was towards aminopenicillins with (50.1%, 95%CI: 24.1–55.9%) and without beta-lactamase inhibitors (40.0%, 95%CI: 33.2–67.1%), followed by quinolones (36.1%, 95%CI: 19.9–52.2%), aminoglycosides (32.7%, 95%CI: 17.4–48.0%), and first and second-generation cephalosporins (31.9%, 95%CI: 17.3–46.7%). Moderate resistance was observed to third and fourth-generation cephalosporins: 29.7%, 95%CI: 15.0–44.4%), tetracyclines (25.3%, 95%CI: 7.6–42.9%), and sulphonamides with folate-reductase inhibitors (24.3%, 95%CI: 9.7–38.9%). Low resistance was recorded against nitrofuran derivatives (8.5%, 95%CI: 0.7–16.7%) and amphenicols (8.2%, 95%CI: 0.2–18.0%). Resistance to carbapenems was observed only in the years 2015, 2016, 2019, and 2021 (2.9%, 95%CI: 0.0–11.9%), while no resistance was ever observed against phosphonic acid derivatives (fosfomycin). The complete list of resistance prevalence per year is presented in Table S2. 

**Figure 2:**
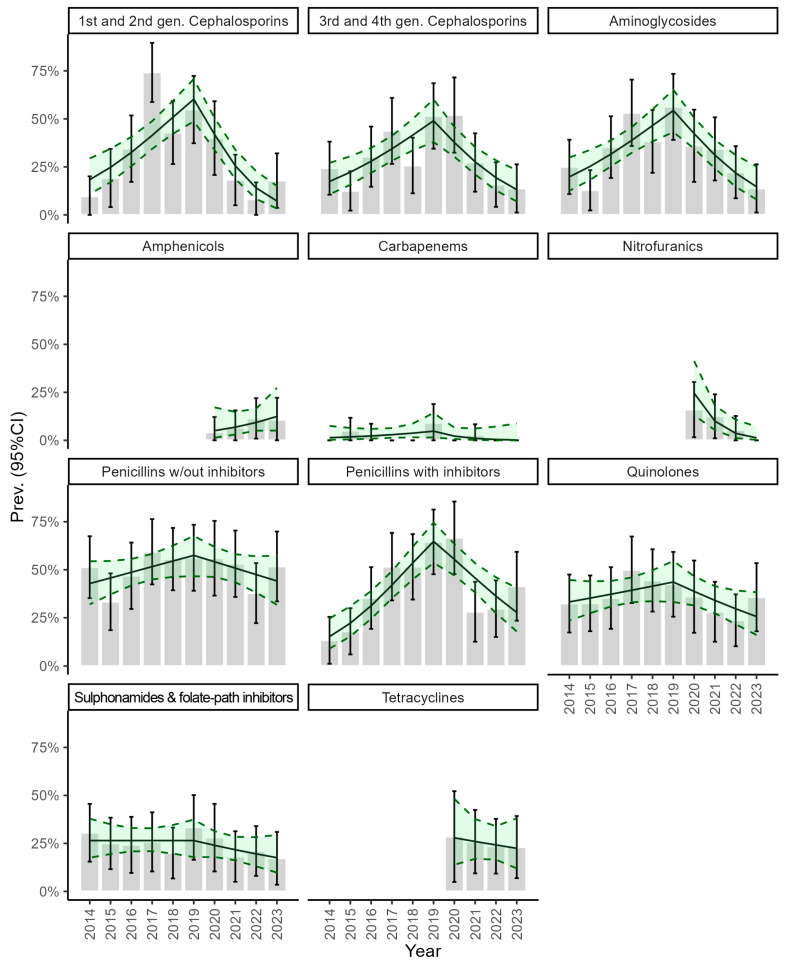
Trend of resistance prevalence by antibacterial class. The bar plots display the prevalence of *E. coli* isolates resistant to each of the eleven tested antibacterial classes, with 95% confidence intervals (error bars). Overlayed, the solid line represents the estimated prevalence trend with its 95% confidence interval (dashed line).

The resistance to aminoglycosides, cephalosporins (all generations), and aminopenicillins with beta-lactamase inhibitors increased significantly until 2019–20, then it decreased significantly. Also, the resistance to quinolones decreased significantly after 2017 (Table 1, Figure 2).

### 3.2. Antimicrobial Resistance to EMA Categories

Age, sex, and the host species were not associated with the resistance to any EMA category. The highest overall resistance was observed to cat. D antibacterials (34.3%, 95%CI: 22.2–46.4%), followed by cat. C (28.0%, 95%CI: 18.6–37.5%), cat. B (23.7%, 95%CI: 13.2–34.2%), and eventually cat. A (14.9%, 95%CI: 8.6–21.3%). No resistance was observed to cat. A during 2022 (Table S3).

In 2019, an inflection of the resistance trend was observed for all EMA categories. Resistance to categories A, B, and C increased significantly until 2019 and decreased significantly afterward (Table 2).

**Figure 3:**
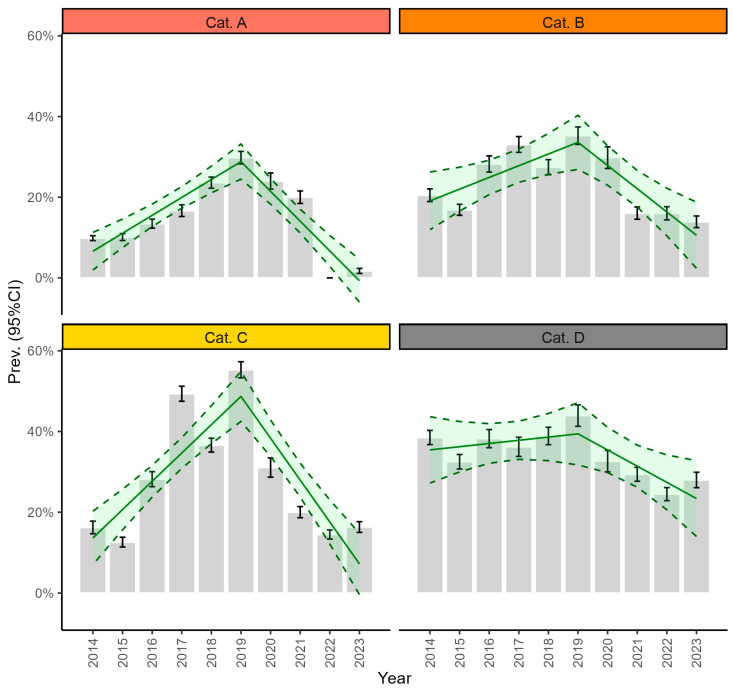
Trend of resistance prevalence by EMA category. The bar plots display the prevalence of *E. coli* isolates resistant to each EMA antibacterial category, with 95% confidence intervals (error bars). Overlayed, the solid line represents the estimated prevalence trend with its 95% confidence interval (dashed line).

Figure 3 displays the trend of the resistance prevalence to each EMA category.

## 4. Discussion

*E. coli* is deemed representative for AMR-monitoring purposes [24] and represents the most frequently isolated species from companion animals with UTI [2,3,4,5,6,7,34,35]. Furthermore, due to the zoonotic potential [13] and bidirectional transmission of AMR between animal and human strains [1], UPEC monitoring is part of the One Health framework. Unfortunately, a nationwide system for monitoring AMR in pets has not yet been implemented, thus AMR surveillance depends on records of individual facilities. Although geographically limited, data on AMR in companion animals gathered by individual facilities are instrumental for complementing national guidelines, supporting the choice of appropriate treatment, and organizing antimicrobial stewardship events for veterinarians [17,26]. Every year, the VTH admits dogs and cats coming from all around the Piedmont region, thus offering useful insights into AMR of UPEC circulating in its catchment area.

Studies on AMR in UPEC have shown an increase in antimicrobial resistance in some countries [34,36], while in others, no significant increase has been observed [36,37,38]. In this study, we observed that the resistance to amphenicols, carbapenems, and nitrofuran derivatives remained essentially constant, which is possibly explained by the limited use of these antibacterials, while the resistance to sulphonamides with folate–reductase inhibitors and tetracyclines steadily decreased since when we started monitoring them, in 2014 and 2020, respectively. On the other hand, the resistance to aminoglycosides, cephalosporins, aminopenicillins, and quinolones has been on the rise since 2014 but, depending on the antimicrobial class, started to decrease in 2018, 2019, or 2020.

Aggregating data by EMA category was crucial to comprehend the evolution of AMR trends. In fact, not only did the resistance level vary among categories, being inversely proportional to the category’s importance for public health, but also resistance to all categories decreased from 2020. The most marked decline was observed for cats. A and C. Carbapenems, tested as a prototype for cat. A, demonstrated exceptionally high efficacy, thus underscoring the absence of discernible pressure on these high-priority drugs from the veterinary community. Conversely, the reduction of resistance to cat. C compounds regarded as antibacterial classes widely used in veterinary practice, i.e., first and second-generation cephalosporins, aminoglycosides, and aminopenicillins with beta-lactamase inhibitors, possibly suggesting a more prudent use of these antibacterials. A limited yet significant reduction was observed in resistance to cat. B antibacterials. whose efficacy was potentially jeopardized by their frequent use in pets. However, it is important to highlight that compounds for exclusive veterinary use exist among fluoroquinolones (cat. B), a circumstance that might have augmented their utilization. The least pronounced decrease was observed for resistance to cat. D, which includes antibacterials of limited importance for public health and thus more easily prescribed to animals.

With these findings, we evidenced that, between January 2014 and November 2023, the trend of AMR of *E. coli* isolated from canine and feline urines has changed. In particular, we observed AMR reduction in several antibacterial classes in the most recent years. These findings are in line with recent reports that showed a decrease in AMR following reduced antibiotic use in Italy [23,39]. Similar findings have been described at the communitarian level by the European Centre for Disease Prevention and Control (ECDC), European Food Safety Authority (EFSA), and European Medicines Agency (EMA) [40]. Several factors could have contributed to reducing AMR. Among them is the enforcement of national and communitarian policies. In fact, during this ten-year period, communitarian legislation fostered the awareness of veterinarians about the threat of AMR [20,41,42], and several measures complemented it at the national level. In Italy, the Legislative Decree 193/2006 was introduced to align European Union (EU) directives on veterinary drugs [43]. In 2019, a decree of the Italian Ministry of Health mandated the use of electronic prescriptions for veterinary drugs and medicated feeds [44]. By 2023, the Legislative Decree 218/2023 enhanced pharmaco-surveillance and established strict guidelines for antibacterials prescription in veterinary practice [45].

Supporting this explanation, the reduction of resistance to the EMA categories mirrored the guidelines regarding antibiotic prescription [41,42]. Indeed, veterinarians should use first-choice or empirical antibacterials based on their experience, with a preference for those in cat. D (e.g., sulphonamides, aminopenicillins without beta-lactamase inhibitors, tetracyclines, etc.). Second-choice antibacterials (drugs included in cat. C group such as aminopenicillins with beta-lactamase inhibitors, macrolides, aminoglycosides, nitrofurans, first and second-generation cephalosporins), should only be used when first-choice options are ineffective or when susceptibility testing indicates their necessity. Third-choice antibacterials, such as quinolones and third and fourth-generation cephalosporins, are included in cat. B and should be reserved for cases where second-choice treatments are ineffective or based on susceptibility testing. Cat. A antibacterials (e.g., carbapenems, tigecycline, etc.) should not be used in any case.

These recommendations should apply in the light of local AMR patterns to ensure safe and effective treatment [26]. Recent studies conducted in Italy were not consistent with the antibacterials to which UPEC was most resistant [4,25]. This study evidenced that, limited to our VTH settings, UTI can be empirically treated with nitrofurantoin and/or sulfamethoxazole-trimethoprim, awaiting AST results. This applies to cats and dogs, as low resistance was observed against cat. D antibacterials in both species.

## 5. Conclusions

The emergence and evolution of antimicrobial resistance pose significant challenges to public health globally. This work delves into the trend analysis of AMR, specifically in UPEC isolated from cats and dogs in the Piedmont region, northwestern Italy, over ten years. The results presented in this study evidenced an inversion of the formerly increasing resistance trend from 2020. The reduction in resistance was more marked for the most restricted antimicrobial classes, based on EMA categorization, and mirrored the guidelines for prudent antimicrobial use in veterinary medicine adopted in the EU and in Italy to fight the spread of AMR.

The results of this study are essential to help the empirical choice of first-line antimicrobial treatment, pending the results of antimicrobial susceptibility testing. Furthermore, they constitute a record of the trend of AMR in a context where there is no organized monitoring system. This could be useful to implement local and regional antimicrobial stewardship interventions.

## Figures and Tables

**Table 1:** Estimates of the slope of resistance prevalence trend by antibacterial class. When no inflection was identified (No), the slope was estimated for the whole 10-year period.

Class	Inflection	OR_prec_ (95%CI)	OR_succ_ (95%CI)
Aminoglycosides	2019	1.37 (1.17–1.62)	0.45 (0.31–0.63)
Aminopenicillins w/out inhibitors	2017	1.22 (0.95–1.57)	0.77 (0.55–1.08)
Aminopenicillins with inhibitors	2020	1.40 (1.22–1.62)	0.42 (0.28–0.62)
Amphenicols	No	0.93 (0.66–1.39)	-
Carbapenems	No	0.93 (0.70–1.20)	-
1st and 2nd gen. Cephalosporins	2019	1.46 (1.23–1.76)	0.32 (0.22–0.48)
3rd and 4th gen. Cephalosporins	2020	1.27 (1.11–1.46)	0.38 (0.24–0.58)
Nitrofuranics	No	0.97 (0.82–1.16)	-
Quinolones	2017	1.28 (0.98–1.67)	0.68 (0.47–0.96)
Sulphonamides and folate-reductase inhibitors	No	0.95 (0.87–1.03)	-
Tetracyclines	No	0.52 (0.39–0.63)	-

**Table 2:** Estimates of the rate of change of resistance prevalence (%) in the years preceding (Coeff_prec_) and succeeding (Coeff_succ_) the inflection, by EMA category.

EMA Category	Inflection	Coeff_prec_ (95%CI)	Coeff_succ_ (95%CI)
A	2019	4.4% (3.0–5.9%)	−11.8% (−14.9–8.8%)
B	2019	2.9% (0.6–5.2%)	−8.7% (−13.4–4.0%)
C	2019	7.0% (4.9–9.1%)	−17.4% (−21.7–13.0%)
D	2019	0.8% (−1.8–3.4%)	−4.8% (−10.2–0.6%)

## Data Availability

Data are available upon request. The datasets presented in this article are not readily available because they are part of an ongoing study.

## Supplementary Materials

The following supporting information can be downloaded at https://www.mdpi.com/article/10.3390/microorganisms12112175/s1, Table S1: Prevalence of antimicrobial resistance by year; Table S2: Prevalence of EMA categories resistance by year; Table S3: Tested antibacterials and their classification.
