# Supplementary material for: Ten-Year Antimicrobial Resistance Trend in Uropathogenic Escherichia coli (UPEC) Isolated from Dogs and Cats Admitted to a Veterinary Teaching Hospital in Italy
Source: Microorganisms. 2024 Oct 29;12(11):2175. doi: 10.3390/microorganisms12112175 (PMC11596680; doi:10.3390/microorganisms12112175)
Supplement: Supplementary file 1 [file microorganisms-12-02175-s001.zip › Table_S1.pdf]

Table S1. Tested antibacterials and their classification. The table displays the list of the antibacterials that *Escherichia coli* isolates were tested for during the ten-year study period.

EUCAST abbr.	Antibacterial	Class	AMEG class	AMEG category	Tested in years
CDR	Cefadroxil	1st generation cephalosporins	Cephalosporins, 1st- and 2nd-generation, and cephamycins	C	2017, 2020
CLE	Cefalexin	1st generation cephalosporins	Cephalosporins, 1st- and 2nd-generation, and cephamycins	C	2014-2023
CLT*	Cefalotin	1st generation cephalosporins	Cephalosporins, 1st- and 2nd-generation, and cephamycins	C	
CZO	Cefazolin	1st generation cephalosporins	Cephalosporins, 1st- and 2nd-generation, and cephamycins	C	2021-2023
CUR	Cefuroxime	2nd generation cephalosporins	Cephalosporins, 1st- and 2nd-generation, and cephamycins	C	2014-2020
CXI	Cefoxitin	2nd generation cephalosporins	Cephalosporins, 1st- and 2nd-generation, and cephamycins	C	2017, 2020-2023
CPE*	Cefoperazone	3rd generation cephalosporins	Cephalosporins, 3rd- and 4th-generation, with the exception of combinations with beta-lactamase inhibitors	B	
CTA	Cefotaxime	3rd generation cephalosporins	Cephalosporins, 3rd- and 4th-generation, with the exception of combinations with beta-lactamase inhibitors	B	2014-2020
CVE*	Cefovecin	3rd generation cephalosporins	Cephalosporins, 3rd- and 4th-generation, with the exception of combinations with beta-lactamase inhibitors	B	2020-2023
CPO	Cefpodoxime	3rd generation cephalosporins	Cephalosporins, 3rd- and 4th-generation, with the exception of combinations with beta-lactamase inhibitors	B	2020
CTZ	Ceftazidime	3rd generation cephalosporins	Cephalosporins, 3rd- and 4th-generation, with the exception of combinations with beta-lactamase inhibitors	B	2014-2020
CTR	Ceftriaxone	3rd generation cephalosporins	Cephalosporins, 3rd- and 4th-generation, with the exception of combinations with beta-lactamase inhibitors	B	2014-2023

EUCAST abbr.	Antibacterial	Class	AMEG class	AMEG category	Tested in years
CEP	Cefepime	4th generation cephalosporins	Cephalosporins, 3rd- and 4th-generation, with the exception of combinations with beta-lactamase inhibitors	B	2014-2023
AMI	Amikacin	Aminoglycosides	Aminoglycosides (except spectinomycin)	C	2014-2023
GEN	Gentamycin	Aminoglycosides	Aminoglycosides (except spectinomycin)	C	2014-2023
NET	Netilmicin	Aminoglycosides	Aminoglycosides (except spectinomycin)	C	2022-2023
TOB	Tobramycin	Aminoglycosides	Aminoglycosides (except spectinomycin)	C	2014-2017, 2022-2023
CHL	Chloramphenicol	Amphenicols	Amphenicols	C	2020-2023
ERT	Ertapenem	Carbapenems	Carbapenems	A	2014-2016
IMI	Imipenem	Carbapenems	Carbapenems	A	2014-2023
MER	Meropenem	Carbapenems	Carbapenems	A	2014-2023
NIT	Nitrofurantoin	Nitrofuran derivatives	Nitrofuran derivatives	D	2020-2023
AMO	Amoxicillin	Penicillins	Aminopenicillins, without beta-lactamase inhibitors	D	2021-2023
AMP	Ampicillin	Penicillins	Aminopenicillins, without beta-lactamase inhibitors	D	2014-2023
BEN	Benzylpenicillin	Penicillins	Natural, narrow-spectrum penicillins (beta-lactamase sensitive penicillins)	D	2017, 2021-2023
PIP	Piperacillin	Penicillins	Carboxypenicillin and ureidopenicillin, including combinations with beta-lactamase inhibitors	A	2014-2023
AMC	Amoxicillin-Clavulanate	Penicillins with beta-lactamase inhibitors	Aminopenicillins, with beta-lactamase inhibitors	C	2014-2023
PIT	Piperacillin-Tazobactam	Penicillins with beta-lactamase inhibitors	Carboxypenicillin and ureidopenicillin, including combinations with beta-lactamase inhibitors	A	2014-2016
FOS	Fosfomycin	Phosphonic acid derivates	Phosphonic acid derivates	A	2014-2016
ENR-CIP	Enrofloxacin/Ciprofloxacin	Quinolones	Quinolones: fluoroquinolones and other quinolones	B	2014-2023
LEV	Levofloxacin	Quinolones	Quinolones: fluoroquinolones and other quinolones	B	2014-2016

EUCAST abbr.	Antibacterial	Class	AMEG class	AMEG category	Tested in years
MAR	Marbofloxacin	Quinolones	Quinolones: fluoroquinolones and other quinolones	B	2015-2023
NOR	Norfloxacin	Quinolones	Quinolones: fluoroquinolones and other quinolones	B	2014-2016
PRA	Pradofloxacin	Quinolones	Quinolones: fluoroquinolones and other quinolones	B	2017, 2020-2023
TRS	Sulfamethoxazole-Trimethoprim	Sulphoonamides, dihydrofolate reductase inhibitors and combinations	Sulphonamides, dihydrofolate reductase inhibitors and combinations	D	2014-2023
TIG	Tigecycline	Tetracyclines	Glycylcyclines	A	2014-2016
DOX	Doxycycline	Tetracyclines	Tetracyclines	D	2022-2023
TET	Tetracycline	Tetracyclines	Tetracyclines	D	2020-2023

* not present into EUCAST official list. Abbreviation created following the EUCAST rules.
