# Supplementary material for: Ten-Year Antimicrobial Resistance Trend in Uropathogenic Escherichia coli (UPEC) Isolated from Dogs and Cats Admitted to a Veterinary Teaching Hospital in Italy
Source: Microorganisms. 2024 Oct 29;12(11):2175. doi: 10.3390/microorganisms12112175 (PMC11596680; doi:10.3390/microorganisms12112175)
Supplement: Supplementary file 1 [file microorganisms-12-02175-s001.zip › Table_S2.pdf]

Table S2. Prevalence of antimicrobial resistance by year. The table displays the number of resistant (Pos.) and susceptible (Neg.) uropathogenic *Escherichia coli* isolates for each antibacterial by year, along with the prevalence proportion (Prev.) and its 95% Wald's confidence interval (95%CI).

Class	Year	Pos.	Tot.	Prev. (95%CI)
Aminoglycosides	2014	9	36	25 (10.9-39.1)
	2015	5	39	12.8 (2.3-23.3)
	2016	12	34	35.3 (19.2-51.4)
	2017	17	32	53.1 (35.8-70.4)
	2018	13	34	38.2 (21.9-54.6)
	2019	18	32	56.3 (39.1-73.4)
	2020	9	25	36 (17.2-54.8)
	2021	11	32	34.4 (17.9-50.8)
	2022	8	36	22.2 (8.6-35.8)
	2023	4	29	13.8 (1.2-26.3)
Aminopenicillins w/out beta-lactamase inhibitors	2014	19	37	51.4 (35.2-67.5)
	2015	13	39	33.3 (18.5-48.1)
	2016	15	32	46.9 (29.6-64.2)
	2017	19	32	59.4 (42.4-76.4)
	2018	20	36	55.6 (39.3-71.8)
	2019	18	32	56.3 (39.1-73.4)
	2020	14	25	56 (36.5-75.5)
	2021	17	32	53.1 (35.8-70.4)
	2022	14	37	37.8 (22.2-53.5)
	2023	15	29	51.7 (33.5-69.9)
Aminopenicillins with beta-lactamase inhibitors	2014	4	30	13.3 (1.2-25.5)
	2015	7	39	17.9 (5.9-30)
	2016	12	34	35.3 (19.2-51.4)
	2017	16	31	51.6 (34-69.2)
	2018	17	33	51.5 (34.5-68.6)
	2019	20	31	64.5 (47.7-81.4)
	2020	16	24	66.7 (47.8-85.5)
	2021	9	32	28.1 (12.5-43.7)
	2022	11	37	29.7 (15-44.5)
	2023	12	29	41.4 (23.5-59.3)
Amphenicols	2020	1	24	4.2 (0-12.2)
	2021	2	30	6.7 (0-15.6)
	2022	4	35	11.4 (0.9-22)
	2023	3	28	10.7 (0-22.2)
Carbapenems	2014	0	37	0.0 (0.0-0.0)
	2015	2	40	5 (0-11.8)
	2016	1	34	2.9 (0-8.6)
	2017	0	32	0.0 (0.0-0.0)
	2018	0	36	0.0 (0.0-0.0)
	2019	3	33	9.1 (0-18.9)
	2020	0	25	0.0 (0.0-0.0)
	2021	1	35	2.9 (0-8.4)
	2022	0	38	0.0 (0.0-0.0)
	2023	0	29	0.0 (0.0-0.0)
1st and 2nd gen. Cephalosporins	2014	3	31	9.7 (0.0-20.1)
	2015	5	26	19.2 (4.1-34.4)

Class	Year	Pos.	Tot.	Prev. (95%CI)
	2016	10	29	34.5 (17.2-51.8)
	2017	23	31	74.2 (58.8-89.6)
	2018	15	35	42.9 (26.5-59.3)
	2019	17	31	54.8 (37.3-72.4)
	2020	10	25	40 (20.8-59.2)
	2021	6	33	18.2 (5-31.3)
	2022	3	37	8.1 (0.0-16.9)
	2023	5	28	17.9 (3.7-32)
3rd and 4th gen. Cephalosporins	2014	9	37	24.3 (10.5-38.1)
	2015	5	40	12.5 (2.3-22.7)
	2016	10	33	30.3 (14.6-46)
	2017	14	32	43.8 (26.6-60.9)
	2018	9	35	25.7 (11.2-40.2)
	2019	17	33	51.5 (34.5-68.6)
	2020	13	25	52 (32.4-71.6)
	2021	9	33	27.3 (12.1-42.5)
	2022	6	38	15.8 (4.2-27.4)
	2023	4	29	13.8 (1.2-26.3)
Nitrofuran derivatives	2020	4	25	16 (1.6-30.4)
	2021	4	32	12.5 (1-24)
	2022	2	37	5.4 (0.0-12.7)
	2023	0	27	0.0 (0.0-0.0)
Quinolones	2014	12	37	32.4 (17.3-47.5)
	2015	13	40	32.5 (18-47)
	2016	12	34	35.3 (19.2-51.4)
	2017	16	32	50 (32.7-67.3)
	2018	16	36	44.4 (28.2-60.7)
	2019	14	33	42.4 (25.6-59.3)
	2020	9	25	36 (17.2-54.8)
	2021	9	32	28.1 (12.5-43.7)
	2022	9	38	23.7 (10.2-37.2)
	2023	10	28	35.7 (18-53.5)
Sulphonamides & folate-reductase inhibitors	2014	11	36	30.6 (15.5-45.6)
	2015	10	40	25 (11.6-38.4)
	2016	8	33	24.2 (9.6-38.9)
	2017	8	31	25.8 (10.4-41.2)
	2018	7	35	20 (6.7-33.3)
	2019	10	30	33.3 (16.5-50.2)
	2020	7	25	28 (10.4-45.6)
	2021	6	33	18.2 (5-31.3)
	2022	8	38	21.1 (8.1-34)
	2023	5	29	17.2 (3.5-31)
Tetracyclines	2020	4	14	28.6 (4.9-52.2)
	2021	7	27	25.9 (9.4-42.5)
	2022	8	34	23.5 (9.3-37.8)
	2023	6	26	23.1 (6.9-39.3)
