# Supplementary material for: Ten-Year Antimicrobial Resistance Trend in Uropathogenic Escherichia coli (UPEC) Isolated from Dogs and Cats Admitted to a Veterinary Teaching Hospital in Italy
Source: Microorganisms. 2024 Oct 29;12(11):2175. doi: 10.3390/microorganisms12112175 (PMC11596680; doi:10.3390/microorganisms12112175)
Supplement: Supplementary file 1 [file microorganisms-12-02175-s001.zip › Table_S3.pdf]

Table S3. Prevalence of EMA categories resistance by year. The table displays the number of analysed uropathogenic *Escherichia coli* isolates for each EMA category by year, along with the prevalence proportion (Prev.) and its 95% Wald's confidence interval (95%CI).

EMA Category	Year	Tot.	Prev. (95%CI)
A	2014	37	9.8 (6.2-13.5)
	2015	40	10.1 (4.7-15.5)
	2016	34	13.4 (6.9-19.9)
	2017	32	16.7 (8.5-24.8)
	2018	36	23.6 (15.3-31.9)
	2019	33	29.8 (20.8-38.8)
	2020	25	24.0 (14.0-34.0)
	2021	35	20.0 (10.8-29.2)
	2022	38	0.0 (0.0-0.0)
	2023	29	1.7 (-1.7-5.1)
B	2014	37	20.5 (10.9-30.0)
	2015	40	16.9 (8.3-25.5)
	2016	34	28.2 (16.5-40.0)
	2017	32	33.1 (22.0-44.2)
	2018	36	27.5 (16.3-38.6)
	2019	33	35.3 (22.8-47.7)
	2020	25	29.8 (16.3-43.3)
	2021	35	16.1 (7.0-25.1)
	2022	38	16 (5.9-26.1)
	2023	29	13.9 (6.1-21.7)
C	2014	37	16.2 (6.7-25.7)
	2015	40	12.6 (5.0-20.2)
	2016	34	28.2 (17.3-39.0)
	2017	32	49.4 (38.8-59.9)
	2018	36	36.6 (26.3-46.9)
	2019	33	55.3 (43.8-66.8)
	2020	25	31.1 (19.2-43.0)
	2021	35	20.0 (11.8-28.2)
	2022	38	14.5 (7.5-21.4)
	2023	29	16.3 (9.2-23.5)
D	2014	37	38.5 (27.8-49.2)
	2015	40	32.5 (21.1-43.9)
	2016	34	38.2 (25.1-51.4)
	2017	32	36.2 (22.7-49.7)
	2018	36	38.9 (25.9-51.9)
	2019	33	43.9 (28.7-59.2)
	2020	25	32.7 (19.3-46.0)
	2021	35	29.4 (19.2-39.6)
	2022	38	24.5 (14.5-34.5)
	2023	29	28.0 (17.7-38.3)
